# External dosimetry audit for quality assurance of carbon‐ion radiation therapy clinical trials

**DOI:** 10.1002/acm2.12465

**Published:** 2018-11-01

**Authors:** Hideyuki Mizuno, Akifumi Fukumura, Nobuyuki Kanematsu, Shunsuke Yonai, Toshiyuki Shirai, Ken Yusa, Toshihiro Yanou, Masaki Suga, Manabu Mizota, Shinichi Minohara, Tatsuaki Kanai, Tadashi Kamada

**Affiliations:** ^1^ National Institute of Radiological Sciences, QST Chiba Japan; ^2^ Gunma University Heavy Ion Medical Center Gunma Japan; ^3^ Hyogo Ion Beam Medical Center Tatsuno Hyōgo Japan; ^4^ Ion Beam Therapy Center SAGA HIMAT Foundation Saga Japan; ^5^ Kanagawa Cancer Center Kanagawa Japan; ^6^ Present address: Tatsuaki Kanai Osaka Heavy Ion Therapy Center Osaka Japan

**Keywords:** carbon‐ion radiation therapy, clinical trial, multicenter dosimetry, onsite dosimetry audit, quality assurance

## Abstract

**Purpose:**

The QA team of the Japan carbon‐ion radiation oncology study group (J‐CROS) was organized in 2015 to enhance confidence in the accuracy of clinical dosimetry and ensure that the facility QA procedures are adequate. The team conducted onsite dosimetry audits in all the carbon‐ion radiation therapy centers in Japan.

**Materials and Methods:**

A special phantom was fabricated for the onsite dosimetry audit. Target volumes such as the GTV, CTV, and PTV were contoured to the obtained CT images, and two plans with different isocenter depths were created. The dose at the isocenter was measured by an ionization chamber, in the onsite audit and compared with the calculated dose.

**Results:**

For all the centers, the average of the percentage ratio between the measured and calculated doses (measured/calculated) was 0.5% (−2.7% to +2.6%) and the standard deviation, 1.7%. In all the centers, the beams were within the set tolerance level of 3%.

**Conclusions:**

The audit demonstrated that the dose at a single point in the water phantom was within tolerance, but it is a big step to say that all doses are correct. In addition, this external dosimetry audit encouraged centers to improve the quality of their dosimetry systems.

## 1 INTRODUCTION

Since 1994, in Japan, carbon‐ion radiation therapy (C‐ion RT) has been used for successfully treating tumors at various sites.^1^ C‐ion beams exhibit increased energy deposition with the penetration depth, up to a sharp maximum at the end of their range, known as the Bragg peak. In addition, they deliver greater mean energy per unit length of their trajectory in the body (linear energy transfer (LET)), compared to proton and photon beams. Tsuji et al. reported that C‐ion RT has clinical advantages over other modalities such as photon IMRT and proton RT.^2^ However, the clinical results reported by most papers were from a limited number of centers. The Japan carbon‐ion radiation oncology study group (J‐CROS) was established in 2014 to obtain clinical evidence through a multicenter C‐ion RT clinical trial.^3^, ^4^, ^5^ Treatment comparability and consistency are crucial for meaningful trial results, particularly, for the absolute dose used in each center or the treatment planning process, from the point of view of medical physics.^6^ Dosimetry audit systems have been established for clinical high‐energy photon and electron beams^7^, ^8^, ^9^, ^10^; however, there was no such audit system for C‐ion RT, in Japan. In 2014, the same year in which J‐CROS was established, medical physicists of J‐CROS institutions conducted dose intercomparison, under the same condition. Physicists from each center brought their dosimeter to a center and conducted the measurements, one after the other, under a single condition. The output difference was within ±0.5% for all the dosimeters.

The J‐CROS QA team was organized in 2015 for the purpose of enhancing confidence in the accuracy of clinical dosimetry and ensuring that the facility QA procedures are adequate. The team, consisting of medical physicists from C‐ion RT centers in Japan, initially conducted surveys in all the centers, using a questionnaire. The questionnaire contained 74 items, including beam calibration and verification (6 items), the irradiation system (18 items), treatment planning (27 items), patient immobilization (3 items), patient setup (11 items), and QA (9 items). The results were shared with all the centers to obtain information on the knowledges and skills of each center. As a result, some centers improved their QA program. Next, the team established the minimum requirements of the medical‐physic items for the center to participate in a J‐CROS clinical trial; it included the following: (a) Establishment of a QA program, (b) National protocol‐based beam calibration and dose specification, (c) Clear specification of the relative biological effectiveness (RBE) model, (d) QA for converting the CT number into the carbon‐ion stopping power ratio, (e) Dose specification for the volume using the standard nomenclature for ICRU reports,^11^, ^12^, ^13^ and (f) Assessment of the patient anatomical and physiological changes during a fraction or over the course of treatment. All these items were checked before credentialing a center for participating in the clinical trials. In the onsite audits, dosimetry audits were performed to assure dose consistency between centers. This paper describes the methodology for external dosimetry audits in carbon facilities, and presents the results of the first round of audits using this methodology.

## 2 MATERIALS AND METHODS

### 2.A Phantom system

**Figure 1:**
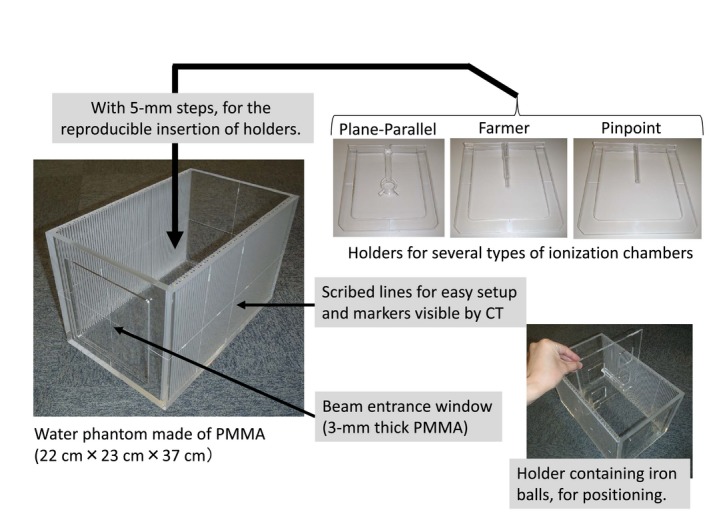
Photographs of the phantom system.

A phantom was designed for performing the onsite dosimetry audit, which involved CT acquisition, treatment planning, position alignment, and ionization dosimetry. The phantom had to be designed such that it can be transported, and accurate dose measurements within a short duration, using ionization chambers, should be possible because of the time constraints imposed by the clinical load. A simple water phantom with PMMA walls was fabricated. A horizontal beam entrance window with a wall thickness of 3 mm was located on the front of the phantom. Grooves were engraved at 5‐mm steps on both sides of the inner wall, along the beam incident axis, to enable the insertion of an ionization chamber holder or alignment jig for positioning. Photographs of the phantom are displayed in Fig. 1. PMMA holders for several types of ionization chambers, such as the plane‐parallel and farmer‐type, were fabricated. The effective point of the detector in the measurement of each ionization chamber followed the IAEA TRS‐398 definition.^14^ The alignment jig included iron balls such that it can be seen in the CT images used for planning as well as the x‐ray images used in the treatment room for patient‐position alignment.

### 2.B Treatment planning

**Figure 2:**
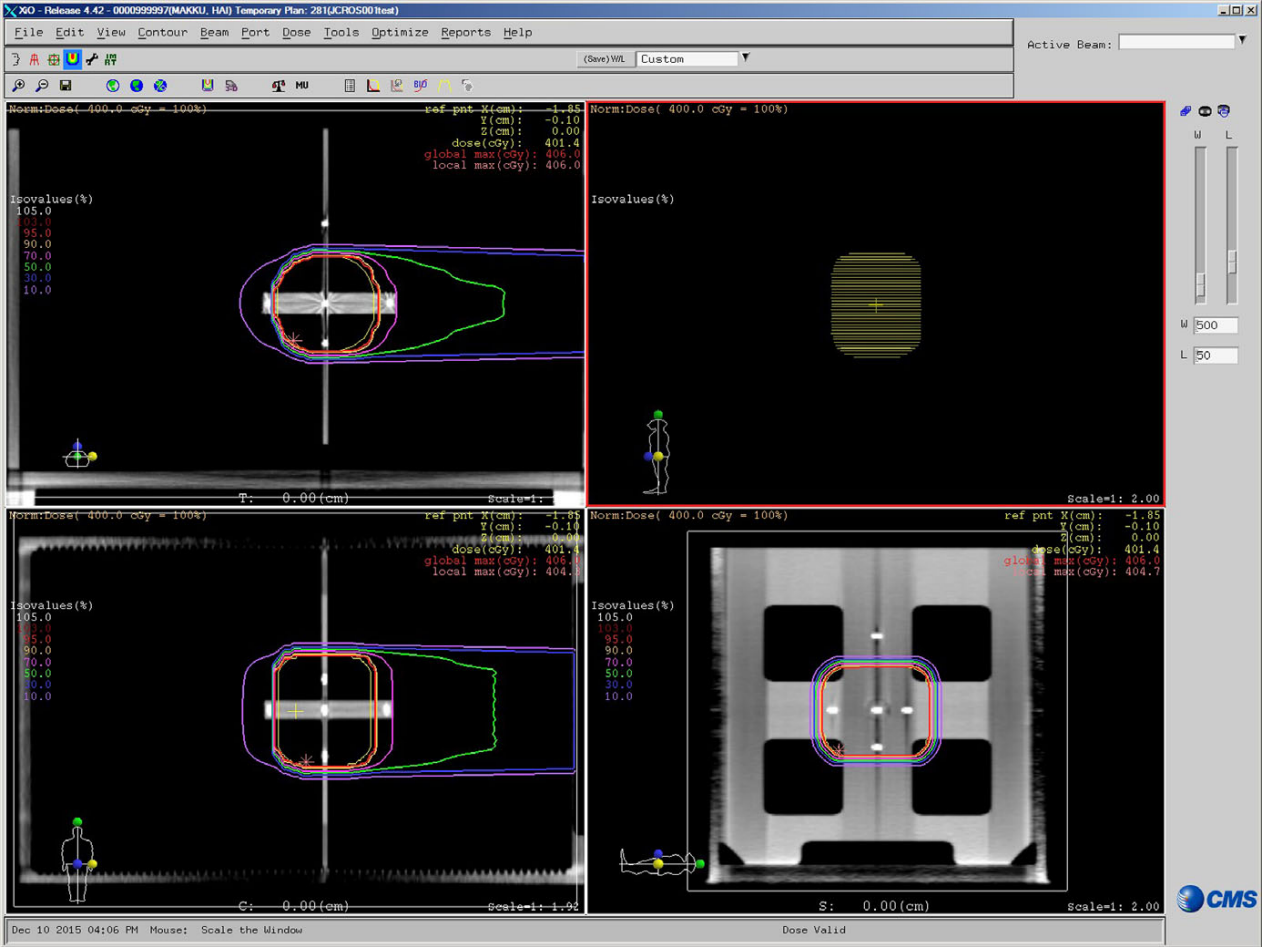
Screen shot of the treatment plan that was generated as per the audit criteria (Scanning beam). Left‐ upper window: Axial CT image, Left‐lower window: Coronal CT image, Right‐upper window: Beam's eye view, Right‐lower window: Sagittal CT image. The yellow contour line shows the PTV shape and CT images with the dose distribution.

Two plans, one using a high‐energy beam and the other using low energy, were used in each center. The actual incident beam energies were approximately 400 and 290 MeV/u, respectively (MeV/u is MeV per nucleon). The isocenter was set to geometric depths of 16.3 and 7.3 cm from the beam entry point. A scribe line identifies the insertion position of the alignment jig at the isocenter depth. The alignment jig has iron balls arranged in a row, corresponding to the beam line. By defining an isocenter on the central iron ball, the planner can easily create a treatment plan with a fixed isocenter. The planner was asked to generate a 3‐cm‐diameter circle gross target volume (GTV) in the isocenter slice and copy it to a length of 3 cm, along the superior–inferior (SI) axis. The margins of the clinical target volume (CTV), internal target volume (ITV), and planning target volume (PTV) were 10 mm (3D), 4 mm (SI direction), and 5 mm (3D), respectively. The prescribed dose was 4 Gy (RBE) at the isocenter; 95% of the prescribed dose should cover the PTV. For calculating the dose distribution, the stopping power ratio of water/PMMA was assigned to the inside or wall of the phantom, respectively. Figure 2 shows a treatment plan, using these criteria.

### 2.C Dosimetry

**Figure 3:**
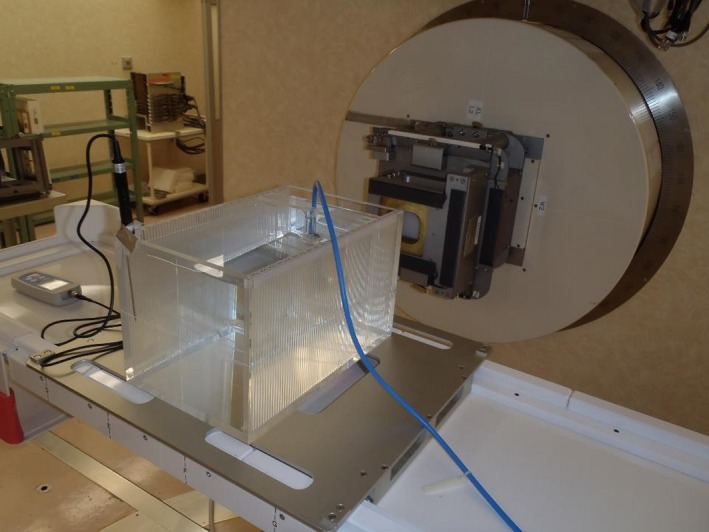
Photograph of the dosimetry with an ionization chamber. The ionization chamber was inserted into the phantom, corresponding to the marked IC position.

The phantom was sent to the center several weeks before the onsite audit. CT images were obtained and treatment plans (for high‐ and low‐energy planning) were created using the same methodology as that used for the patients. The J‐CROS QA team then visited the center with a calibrated dosimeter, thermometer, and barometer. The same tools were used in all the audits conducted in this study. For the dosimeter, an Advanced Markus chamber (TN34045, PTW‐Freiburg, Freiburg, Germany) in combination with an electrometer (PC‐electrometer, Sun Nuclear, Melbourne, FL, USA) was used. The set up and alignment of the phantom was performed by local radiation technologists, similar to that for a patient. Alignment was carried out using 2D‐based procedures with orthogonal x‐ray images and DRR images, for all the centers. The precision of the setup was verified using a line marker on the outside wall of the phantom, which corresponded to the correct position of the ionization chamber. All the centers could successfully locate the phantom in the correct position within a precision of 1 mm. After aligning the phantom, the alignment jig was removed, and a chamber holder equipped with the ionization chamber was inserted (Fig. 3). Carbon‐ion beam irradiation was performed, according to the irradiation parameters determined by the treatment planning system. To check the reproducibility, the same irradiation was repeated several times. The reading of the ionization chamber was converted into the absorbed dose using the Japanese reference dosimetry code of practice (standard dosimetry of the absorbed dose to water in external beam radiotherapy),^15^ based on the IAEA TRS 398. The obtained dose data were compared with the output of the physical dose treatment plan. All five centers that had performed C‐ion RT in Japan, in 2016, participated in this dosimetry audit.

## 3 RESULTS

### 3.A Facility specifications

The general data obtained from the questionnaire, including the beam specifications, treatment planning system, and the reference dosimeters used in the center are summarized in Table 1.

**Table 1:** List of tested centers and their general specifications

Centers	Gunma University Heavy Ion Medical Center	Hyogo Ion Beam Medical Center	Research Center for Charged Particle Therapy, NIRS (Passive)	Research Center for Charged Particle Therapy, NIRS (Scanning)	Ion Beam Therapy Center, SAGA HIMAT Foundation	Kanagawa Cancer Center, iROCK
Date of audit	2015/10/28	2015/11/24	2015/12/11	2015/12/12	2016/1/15	2016/3/18
Irradiation method	Passive	Passive	Passive	Scanning	Passive	Scanning
Maximum field size	15 × 15 cm	15 × 15 cm	22 × 15 cm	22 × 22 cm	16 cm *ϕ*	22 × 22 cm
Treatment beam maximum range	25.9 cm	17.5 cm	28.7 cm	30.0 cm	24.3 cm	27.0 cm
Maximum SOBP width	12 cm	14 cm	15 cm	30 cm	12 cm	30 cm
Average spot size (1SD)	–	–	–	2.5 mm	–	2.5 mm
Treatment planning system	XiO‐N (Mitsubishi electronic)	XiO‐M (Mitsubishi electric)	XiO‐N (Mitsubishi electronic)	XiDose (NIRS, Elekta)	XiO‐N (Mitsubishi electronic)	Monaco‐I (Elekta)
Conversion from CT value to stopping power ratio	Polybinary tissue model	Polybinary tissue model	Polybinary tissue model	Polybinary tissue model	Polybinary tissue model	Polybinary tissue model
Determination of dose per monitor unit	Measurement	Measurement	Measurement or calculation from semi‐empirical formula	TPS calculation	Measurement	TPS calculation
Ionization chamber type used for the absolute dose calibrations	Thimble type chamber	Thimble type chamber	Plane‐parallel chamber	Plane‐parallel chamber	Thimble type chamber	Plane‐parallel chamber

Most items were equivalent in all the centers, including the maximum field size, maximum range of the treatment beam (excluding one center), and the procedure for converting the CT value into the stopping power ratio. The maximum range of the treatment beam is valid for a specific field size, such as a beam with a diameter of 10 cm and not necessarily the maximum field size. Several clear differences were found between the passive and scanning irradiation methods, such as the maximum spread‐out Bragg peak (SOBP) width and the method of determining the dose per monitor unit. As the ionization chamber for reference dose calibration, 50% of the centers used cylindrical chambers (PTW type 30013), whereas the others used plane‐parallel chambers (PTW type 23343 and type 34045). IAEA TRS 398 accepts both types of chambers as reference absorbed dose detectors. Although the calibration factor uncertainty with a cylindrical chamber is lower than of a plane‐parallel chamber, its use is recommended, only if the dose gradient within the chamber volume is low.

### 3.B Dosimetry results

The obtained results are shown in Table 2. The average of the percentage ratio between the measured and calculated doses (measured/calculated) was 0.5% (−2.7% to +2.6%) and the standard deviation, 1.7%. Currently, there is no established agreement criteria between the measured and calculated absorbed doses for passive and scanning C‐ion beams. The European organization for research and treatment of cancer‐radiation oncology group (EORTCROG) has adopted 3% as the optimal limit for the beam output audit of photon and electron beams.^16^ Abletinger et al.^17^ suggested that the mean value of the deviation between the measured and calculated doses should be less than 3%, while the maximum deviation should be less than 5% for the target volume in a homogeneous medium, in the dosimetry auditing of light‐ion beam therapy. In this case, the tolerance level was set within 3%. All the beams of all the centers were within this tolerance level.

**Table 2:** Summary of the dosimetry audit results. The SD value next to the result value of each facility is the standard deviation of several iterative measurements in the ionization chamber. The SD value in the ‘total average’ cell is the standard deviation of all the various values of the facility

Facility^a^	Plan 1 (high‐energy) difference between the measured and calculated doses	Plan 2 (low‐energy) difference between the measured and calculated doses
A	+1.0% (SD = 0.2%)	+0.8% (SD = 0.2%)
B	+2.1% (SD < 0.1%)	+2.6% (SD < 0.1%)
C	+1.4% (SD = 0.1%)	+1.2% (SD < 0.1%)
D	−1.0% (SD = 0.1%)	+1.1% (SD < 0.1%)
E	−2.7% (SD < 0.1%)	−1.4% (SD = 0.1%)
F	0.0% (SD = 0.1%)	−0.1% (SD = 0.1%)
Average	0.1%	0.7%
	Total average 0.5% (SD = 1.7%)	

a One of the five participating centers operates both passive and scanning beam facilities; the test result of the scanning beam of the center was tabulated as an independent facility.

## 4 DISCUSSION

The results were satisfactory because none of the beams exceeded the tolerance levels. This audit has demonstrated that the dose at a single point in the water phantom is within tolerance; it is a big step to say that all the doses are correct. In addition, this external audit has encouraged the centers to improve the quality of their dosimetry systems.

Center C improved the dosimetry accuracy, based on the results of this test. The thermometer and barometer had not been calibrated because they were mounted on an irradiation system and could not be easily calibrated independently; however, they were finally calibrated with traceability to the national standards. This altered the reference dose by approximately 0.8%, resulting in a decrease in the difference between the measured and calculated doses, in the test results of this study. The corrected results of center C were +0.6% and +0.4% for plans 1 and 2, respectively.

In center E, where the difference in plan 1 exceeded 2%, an old‐type ionization chamber (TN23343, PTW‐Freiburg, Freiburg, Germany) was used as the reference dosimeter. This reference dosimeter was updated to a new type (TN34045, PTW‐Freiburg), based on the results of this test. According to the measurement results, the absolute dose varied from +(0.6–0.9)%, depending upon the irradiation conditions. Using the average value of +0.7%, the corrected results of center E were −2.0% and −0.7%, respectively.

In view of the above, it is established that dose auditing improves the quality of the dose precision applied in clinical trial.

In external dosimetry audits, there are several reports in the case of conventional RT, such as photon therapy; however, they are rarely reported for particle beam therapy, particularly for C‐ion RT. Ableitinger et al.^17^ reported end‐to‐end testing by the mailing method, using alanine dosimeters. However, in the case of alanine dosimeters, the dose rate and LET effects of the scanning beam must be considered, when analyzing the detector response. In addition, it is difficult to perform at a dose equivalent to the protocol defined for a multicenter clinical trial. As the alanine dosimeter requires a dose irradiation of 10 Gy or more for good reproducibility, it is not suitable for the verification of the treatment plan, for credentialing. As there are five C‐ion RT centers in Japan, it is possible to perform onsite dosimetry audit; auditing can be done using ionization‐chamber dosimetry, which is a high‐precision standard dosimetry tool. Hence, in this study, we adopted a tolerance criterion of 3% and succeeded in assuring precise dosage.

In addition to physical dose matching, the RBE is another important value to be checked, among the centers participating in the clinical trials for C‐ion RT. Using the treatment planning system of each center, the calculated RBE values for different conditions such as the incident beam energy, depth, and prescription dose were compared. The detailed description of the results is not within the scope of this work; however, the results were comparable for all the centers.

## 5 CONCLUSION

The J‐CROS QA team conducted onsite dosimetry audits at all the C‐ion RT centers in Japan. The audits were performed using two treatment plans, and the results were within the tolerance levels (<±3%), for all the centers. The activities of this QA team will continue and will be applied to new treatment centers in Japan, where C‐ion RT is initiated.

## CONFLICT OF INTEREST

The authors declare no conflicts of interest.
